# Lace Up and Mindfulness: A Randomized Controlled Trial Intervention to Reduce Emotional Eating, Anxiety, and Sleep Disturbances in Latinx and Black Youth

**DOI:** 10.3390/children10030538

**Published:** 2023-03-10

**Authors:** Norma Olvera, Sascha Hein, Molly Matthews-Ewald, Rongfang Zhang, Rhonda Scherer

**Affiliations:** 1 Department of Psychological, Health, and Learning Sciences, University of Houston, Houston, TX 77204, USA; 2 Department of Education and Psychology, Free University Berlin, 14195 Berlin, Germany; 3 Creative Research Solutions, LLC, Snellville, GA 30078, USA; 4 Sports & Fitness, University of Houston-Downtown, Houston, TX 77002, USA

**Keywords:** mindfulness, eating, health outcomes, anxiety, randomized controlled trial

## Abstract

This study assessed the effects of a 12-week afterschool mindfulness-based diet and exercise intervention on mental and physical health in Latinx and Black youth. One hundred forty-eight boys and girls (average age = 10.1 years, SD = 1.3 years; 52% girls; 72.3% Latinx) were randomized to either the experimental group (*n* = 80) or the control group (*n* = 68). The experimental group participants engaged in fitness yoga, kickboxing, and/or spinning sessions, and mindfulness practices (e.g., breathing, meditation, and mindful eating) twice per week for 12 weeks. The control group participants engaged in a recreational play session once per week for 12 weeks. All participants completed surveys (demographics, acculturation, anxiety, emotional eating, sleep, and food intake) and had their height, weight, and percent body fat measured pre- and post-intervention. Participants wore an accelerometer for 7 days pre- and post-intervention. Repeated measures analysis of covariance indicated that the experimental group participants reported lower scores in emotional eating, anxiety, and sleep latency post-intervention compared to the control group participants. Conversely, no significant differences were observed in physical activity between the experimental and control group participants post-intervention. These findings indicate that a mindfulness-based intervention has a positive effect on emotional eating, anxiety, and sleep latency among youth of color.

## 1. Introduction

Childhood obesity remains a persistent health problem in the United States. According to national data, 19.3% of American children (ages 6–11) and 20.9% of adolescents (ages 12–19) are classified as obese [1]. Childhood obesity is disproportionately affecting children and adolescents of color compared to their White peers. Specifically, 28% of Mexican American and 23% of African American children between the ages of 6 and 11 have been found to have obesity compared to 16% of White children. This discrepancy persists throughout childhood and into young adulthood. More specifically, even higher disproportional obesity rates are reported in adolescents and young adults (ages 12–19), with 31% of Mexican American and 28% of African American adolescents and young adults having obesity compared to 21% of their White counterparts [1]. The physical and psychological consequences of childhood obesity are well documented. Children and adolescents with obesity are more likely to engage in maladaptive eating practices such as emotional eating [2] and overeating [3]; engage in lower levels of physical activity [4]; report higher rates of both anxiety and depression [5]; and sleep disturbances [6] compared to their counterparts of normal weight. Because of the physical and psychological consequences of obesity and high obesity prevalence among children and adolescents, particularly youth of color, the development of effective interventions to prevent and treat obesity among youth is crucial.

In general, childhood and adolescent obesity prevention and treatment interventions have focused on diet only, physical activity only, or a combination of diet and physical activity, and have yielded unsatisfactory or mixed results. For example, Brown and colleagues [7] conducted a meta-analysis review of 153 randomized controlled trials (RCTs) of obesity interventions that included children and adolescents (ages 0–18 years; 85 studies focused on children ages 6–12 years). The results from this meta-analysis suggest that obesity interventions that incorporated only diet reduction or only increased physical activity were not effective in lowering standardized body mass index (BMI) z-scores. Similarly, other meta-analyses have shown no statistically significant BMI and BMI z-score reductions in children and adolescents after participation in nutrition and physical activity interventions [8,9].

Given the limited effectiveness of childhood obesity interventions relying primarily on improving the quality of dietary intake and physical activity, alternative approaches to developing obesity programs have been suggested. Based on Marks’s circle of discontent theory [10], obesity programs might focus on addressing psychological pathways linking obesity and negative affect. Furthermore, in developing obesity programs, Rosenbaum and White [11] call for a more complete biopsychosocial approach that includes the physiological implications of the negative affect that can be associated with obesity. Emotional eating has been identified as a way that some individuals mitigate negative affect and is strongly and positively associated with both weight gain [12,13,14], and a higher percentage of body fat in youth [15]. Therefore, focusing on decreasing emotional eating episodes may curb the consumption of energy-dense foods, thereby reducing the likelihood of excess weight gain. Emotional eating is defined as either eating in response to emotional cues (e.g., boredom, anxiety or fear, sadness, or depression) [16] or eating in an unhealthy manner to alleviate negative emotions [17].

Mindfulness has emerged as an approach that can improve emotional eating regulation. Mindfulness, according to Kabat-Zinn [18] (p. 4), is defined as ‘paying attention in a particular way: On purpose, in the present moment, and nonjudgmentally.’ Mindfulness is often further described as being self-aware, focusing on the present moment, and acknowledging feelings, thoughts, and body sensations in a non-judgmental manner [19]. Interventions including mindful eating practices (e.g., eating slowly) have been effective in decreasing engagement in emotional eating because they promote emotion regulation designed to reduce caloric intake [20] and sugar and fat consumption [21]. Moreover, mindful eating practices encourage individuals to pay close attention to body-related sensations in response to the foods they consume as well as thoughts they have about food [22]. Mindful eating has also been used to increase dietary self-control to suppress short-term impulses to eat with the aim of pursuing long-term weight goals [23]. Some studies have also shown that mindfulness meditation interventions reduce emotional eating in college students [24] and food cravings and overeating in women with overweight [25]. However, no studies have been conducted to assess the effects of mindfulness practices such as meditation and mindful eating on emotional eating among youth of color.

Compared to their impact on emotional eating, the effects of mindfulness practices on reducing anxiety and sleep disturbances have been more extensively investigated [5,6]. For instance, Eberth and Sedlmeier [26] reviewed 49 studies involving different types of meditation practices. The results from this review indicated that there is a strong association between mindfulness practices and stress and anxiety reduction and lower negative emotions. Dunning and colleagues [27] reviewed 33 RCTs and found significant positive effects of mindfulness-based interventions on depression and anxiety among children and adolescents. Similarly, intentional breathing exercises have demonstrated improvement in sustained attention, affect, and cortisol levels [28]. In sum, mindful stress reduction practices including meditation and intentional breathing exercises have been found to reduce mental distress in youth after an intervention. In addition, integrating mindfulness practices with physical activities such as yoga has shown positive effects on mental health [29]. Studies indicate that children who engage in yoga may experience a reduction in stress or anxiety, enhancement in mood [30,31], and improved breathing efficiency [32]. Furthermore, school-based yoga-type programs have shown decreases in anxiety and depression scores among children immediately after they participated in these programs [33,34,35]. Kennedy and Resnick [29] also suggest that integrating mindfulness with exercise is one way to initiate exercise and improve self-efficacy.

Mindfulness–exercise practices have also been recognized to be beneficial in lowering sleep disturbances (e.g., quality, duration, and latency) in adult women (e.g., via a mobile app) [36]. Limited research has been conducted on the effects of mindfulness–exercise-based interventions on youth’s sleep disturbances [6], and these studies appear to have had mixed results. Some research has shown that mindfulness–exercise-based interventions improved global sleep quality, sleep onset latency, and daytime sleepiness among adolescents [37]. In addition, Bei and colleagues [38] reported that sleep education and mindfulness training (e.g., meditation) had beneficial effects on the sleep quality of girls aged 13–15 years. By contrast, Sibinga et al. [39] found that although a school-based mindfulness intervention was associated with reduced anxiety and rumination, it did not have an impact on sleep quality among high school boys (with 95% of the sample being African American). Taken together, these studies suggest that promoting mindfulness practices (e.g., meditation, breathing, and yoga) and exercise has positive effects on participants’ physical and mental health.

Despite the positive effects of mindfulness, very few mindfulness interventions have included youth of color. Thus, the purpose of this study was to test the efficacy of a 12-week mindfulness diet- and exercise-based intervention named Lace Up and Mindfulness (LUAM) in reducing emotional eating, reducing anxiety, and improving sleep quality among Latinx and Black youth using an RCT approach. It was hypothesized that experimental group participants would exhibit lower anxiety and emotional eating scores, and increased sleep quality compared to the control group participants after completing a 12-week intervention.

## 2. Materials and Methods

### 2.1. Participants

The sample consisted of 148 children (52% girls [*n* = 77]; mean age = 10.11 years, SD = 1.3 years; 72.3% Latinx, 18.9% Black, 8.8% multiracial [ClinicalTrials.gov identifier is NCT03885115]). To participate in this study, children had to be: (1) between 9 and14 years old; (2) of Latinx or Black descent; (3) without a physical disability (e.g., inability to walk) or medical conditions (e.g., heart condition) that may interfere with their participation in the exercise part of the intervention; and (4) willing to participate in pre- and post-intervention measurement sessions. Children, regardless of their weight status, were encouraged to participate. Recruitment efforts were conducted at seven elementary schools with a >80% enrollment of Latinx or Black children from a major southwest city independent school district. With the permission of school administrators, research assistants employed several recruitment strategies: (a) sending study flyers home with children; (b) informing caregivers (mainly mothers) and children about the study at school events such as health fairs, parent-teacher organization meetings, and celebrations; and (c) referrals from school nurses and teachers to encourage eligible families to participate in this study. The university’s Institutional Review Board approved the study protocol.

### 2.2. Procedures

The research team invited interested caregivers and their children to attend a study orientation session at the child’s school grounds. During the study orientation, research assistants provided the caregiver and child with more detailed information about the study description, expectations, time commitment, randomization procedures, and eligibility requirements. At this time, potential participants also had an opportunity to ask questions. At the end of each orientation, if potential participants were still interested in participating in this study, the caregiver signed an informed consent form for their participation and a permission form for their child. Children also signed an assent form if they agreed to participate. Subsequently, research assistants scheduled parents and children for two (2) 60 min baseline measurement sessions conducted at the child’s school. For this study, only child data collection and analyses are presented since we explored the impact of an intervention on children’s mental and physical health.

During the first baseline measurement session, after a brief instruction section, children completed a series of surveys and had their height, weight, and percent body fat recorded privately. At the end of this first baseline measurement session, each child received an accelerometer (ActiGraph wGT3X-BT model), with instructions on how to wear it for 7 days. Within one (1) week, research assistants scheduled a second baseline measurement session where participants finished completing the surveys, returned the accelerometers, and received information on the next steps of the study. Research assistants followed similar measurement procedures during the post-intervention measurement session (12 weeks after the pre-intervention assessment). After the completion of baseline data collection, the assignment to either the experimental group (EG) or control group (CG) was conducted based on a simple (i.e., unstratified) random sampling procedure. A study researcher used a randomization table to randomly assign the child ID number to one of the study conditions and concealed this process from all study researchers, project coordinators, research assistants, and instructors. Then, the researcher informed the project coordinator of the participants’ assigned study condition, so they could subsequently notify families of their assigned study condition. In the case of a family that had two children eligible to participate in this study, both children were assigned to the same treatment condition to circumvent treatment diffusion across EG and CG participants.

### 2.3. Intervention Description

#### 2.3.1. Experimental Group (EG)

The EG participants took part in the LUAM intervention aimed to reduce emotional eating, anxiety, and sleep disturbances through mindfulness practices. Specifically, the LUAM intervention consisted of a 12-week after-school program that included two group sessions per week of either one (1) 60-min kickboxing or spinning session and one (1) 60-min fitness yoga session with the inclusion of mindfulness practices at each exercise session. Certified fitness instructors led each exercise session, consisting of a 5-min warm-up period, 40-min kickboxing/spinning or fitness yoga session, 10-min mindfulness practices and cool-down period, and a 5-min bathroom and water break. In this study, kickboxing included repetitive rapid movement with hands (e.g., throwing punches) and feet (e.g., kicking) with increasing intensity. Kickboxing was chosen as one type of exercise as it provides an aerobic workout, which is beneficial for burning calories, and improving cardiovascular fitness. Kickboxing incorporates martial art techniques and boxing skills accompanied by popular music and is aimed at increasing energy expenditure while strengthening core muscles [40]. During the spinning sessions, the EG participants used a stationary bicycle with a weighted flywheel to increase or decrease resistance while pedaling. The spinning focused on increasing endurance, strength, and intensity. Spinning has been shown to increase cardiovascular endurance [41]. The fitness yoga sessions incorporated both strengthening and stretching activities in a fast-paced environment, increasing energy expenditure while promoting breathing control and meditation. Fitness yoga is reported to benefit children’s physical and mental health [42].

Additionally, the EG participants engaged in intentional mindfulness practices during and after each exercise session. Mindfulness practices focused on guided intentional focus or meditation, sustained attention, breathing practices, body awareness, and mindful eating [43]. For example, children engaged in guided meditation and breathing practices during yoga and kickboxing/spinning. Further, children engaged in breathing activities such as butterfly breath and hot chocolate breath. In the butterfly breath activity, the child used either their hands, arms, or legs to move or flap as they would breathe in and out. For the hot chocolate breath activity, the child would pretend to be freezing and have a hot chocolate in their hands. The child would blow on hot chocolate to cool it down. Furthermore, children were asked to practice breathing and meditation any time they felt afraid, anxious, or upset.

The EG participants also engaged in several mindful eating activities while consuming a healthy snack and water. For instance, children were encouraged to eat food items slowly and with their eyes closed to become more aware of food traits through other senses, such as smell, touch, and taste, while noting thoughts, feelings, and physical responses to that food. Another example of a mindful eating activity involved children focusing on body awareness with an emphasis on recognizing eating cues for hunger or emotional factors. Those in the EG also participated in food demonstrations of healthy snacks (e.g., parfaits and smoothies) that they could make on their own at home. The combined exercise and eating snack sessions lasted 1.5 h per week. The EG participants received a LUAM t-shirt at the end of the intervention.

#### 2.3.2. Control Group (CG)

CG participants attended weekly 1.5 h recreational play sessions for 12 weeks. These sessions included several recreational games, such as “Tic Tac Toe” (relay game) and “Pac-Man” (tagging), and playing sports such as soccer or basketball. Each week, research assistants provided CG participants with specific options for games or sports to play. Children selected which game or sport to play for the first 30 min of the session and then switched activities for another 30 min if they wished to change. If they did not wish to change, they could play the same game or sport for another 30 min. CG participants received a healthy snack and water at the end of each session and a t-shirt at the end of the program.

#### 2.3.3. Setting and Safety

Both the EG and CG group sessions were implemented for different cohorts of participants at the children’s school facilities such as a school gym, an empty classroom dedicated to our study, or school green areas. The EG and CG group sessions were typically held between 4:00 and 5:30 p.m. or 4:30–6:00 p.m. (depending on the principal’s approval) during 2017–2019 before the COVID-19 pandemic. Participants’ safety was paramount in this study. Several safety guidelines were followed to minimize participants’ risk of injury. For instance, before each exercise session (experimental and control), the instructor reminded participants to check the laces of their tennis shoes to make sure they were tied. During the EG sessions, participants were encouraged to breathe and exercise at a safe and effective pace that they could maintain. Modifications were provided to ensure safe engagement in the activities and to accommodate varied fitness levels. Instructors were required to complete the American Red Cross CPR/First Aid/AED training and have a readily available first aid kit.

### 2.4. Measures

Except for demographic and acculturation data collected only at baseline, data were collected at two time points: (1) baseline (T1) assessment before group assignment randomization, and (2) post-intervention (T2) assessment immediately after participation in the 12-week intervention.

#### 2.4.1. Demographic Characteristics and Acculturation

The demographic questionnaire included 12 items that assessed age, gender, grade, family structure (i.e., number of family members living in the same household), birthplace (e.g., the United States, Mexico, Central America, etc.), and access to fitness equipment (e.g., bike). All participants completed the 12-item Short Acculturation Scale for Hispanic Youth [44]. This instrument assessed acculturation to mainstream culture in terms of language use/proficiency (9 items, e.g., “What language do you usually speak at home?”), and social relations (3 items, e.g., “Your close friends are…”). All participants answered these acculturation questions on a 5-point Likert-type scale ranging from 1 (only Spanish) to 5 (only English) and from 1 (all Hispanic) to 5 (all non-Hispanic), respectively. A sum score greater than or equal to 30 indicated high acculturation and a sum score of less than 30 designated low acculturation. The internal consistency of participants’ acculturation scale scores was adequate for Latinx participants (Cronbach’s α = 0.88) and for Black participants (Cronbach’s α = 0.79).

#### 2.4.2. Weight Status and Percent Body Fat

Trained research assistants measured each participant’s body height using a Seca 213 stadiometer (Hamburg, Germany) and rounded to the nearest 0.1 cm, and body weight using a scale (Tanita SC-331S, Tokyo, Japan), rounded to the nearest 0.1 kg. Participants were weighed without shoes or heavy clothing. Based on body height and weight, research assistants computed the body mass index using Quetelet’s index [body weight (kg)/height (m^2^)]. Obesity status was calculated using BMI values for the age and sex-specific percentiles according to the Centers for Disease Control and Prevention (CDC) guidelines [45]. Using CDC guidelines, participants were classified as being underweight with a BMI < 5th percentile; healthy weight with a BMI in the 5th–84th percentile; overweight with a BMI in the 85th–94th percentile; and obese with BMI ≥ 95th percentile. The Tanita scale was also used to assess percent body fat (%BF).

#### 2.4.3. Emotional Eating

Emotional eating was assessed using the McKnight Risk Factor Survey IV [46] emotional eating subscale. The emotional eating score was computed by averaging responses to six items. Three out of the six items had questions regarding eating less such as, “In the past month, how often did you eat less than usual (1) when you were bored? (2) when you try to feel better about yourself?; and (3) when you were upset?” The other three items included questions involving eating more such as, “In the past month, how often did you eat more than usual when you were bored, trying to feel better about yourself, and when you were upset?” Items from this subscale were rated using a 5-point Likert scale (1 = never to 5 = always). In this study, the internal consistency (Cronbach’s α) of the emotional eating subscale for the pre- and post-assessments was good (0.86 and 0.87, respectively).

#### 2.4.4. Multidimensional Anxiety Scale for Children, 2nd Edition (MASC-2™)

The MASC-2™ is a comprehensive measure of anxiety-related symptoms in youth aged 8 to 19 years [47]. This 50-item measure assesses a broad range of emotional, physical, cognitive, and behavioral symptoms of anxiety. Participants answered items on a 4-point Likert scale, ranging from 0 (never true about me) to 3 (often true about me). The questionnaire yields raw scores and standardized T-scores of the overall degree of self-reported total anxiety, six anxiety scales, and four subscales. These anxiety scales assess separation anxiety/phobias, generalized anxiety disorder, social anxiety (humiliation/rejection and performance fears subscales), obsessions and compulsions, physical symptoms (panic and tense/restless subscales), and harm avoidance. Following the MASC-2™ scoring guidelines, T-scores were used for analyses in the current study, as they are standard scores using the MASC-2™ profile for boys and girls. The MASC-2™ is recognized as a valid and reliable anxiety scale, with excellent internal consistency (Cronbach’s α = 0.92 for the total score) [48]. In the current sample, the internal consistency of the MASC-2™ total score was excellent for both pre- and post-intervention assessments (Cronbach’s α = 0.94).

#### 2.4.5. Pittsburg Sleep Quality Index (PSQI)

The PSQI is a 19-item self-report questionnaire composed of seven subscales: subjective sleep quality, sleep latency, sleep duration, habitual sleep efficiency, sleep disturbances, use of sleep medication, and daytime drowsiness [49]. The present study focused on three sleep quality subscales (sleep duration, sleep efficiency, and sleep latency) as suggested by previous research [50]. Duration is a measure of total sleep hours at night per day. Efficiency refers to the percentage of total time in bed spent in sleep. Latency is a computed Likert rating (scores of 0 to 3) based on two indicators of how quickly a participant falls asleep (where higher scores indicate more difficulty in falling asleep). A global PSQI score ranged from 0 to 21. Global scores lower than 5 indicate healthy sleep quality, whereas scores greater than or equal to 6 indicate worse sleep quality. The internal consistency of the PSQI was reported by Ranit et al. [51] as acceptable (Cronbach’s α = 0.73). In this study, the internal consistency of the PSQI was acceptable for both the pre-intervention assessment (Cronbach’s α = 0.74) and post-intervention assessment (Cronbach’s α = 0.69).

#### 2.4.6. Physical Activity

Pre-to-post-intervention changes in physical activity (i.e., duration, frequency, and intensity) were assessed using an accelerometer (ActiGraph wGT3X-BT model). Research assistants provided participants with instructions (both written and oral) on how to wear an accelerometer fastened to a belt and placed on the right hip for 7 days (including sleep time). A valid assessment was determined by a minimum of 8 h of wear time for at least 4 days (2 weekdays and 2 weekend days). A one-minute epoch was the amount of time over which activity counts were integrated and recorded. The algorithm utilized to identify non-wear time in the ActiGraph device included intervals of at least 60 consecutive minutes of zero activity counts between 1 and 100 counts. Following the pre- and post-data collection, the accelerometer data were downloaded and analyzed using the ActiLife software to determine participants’ physical activity intensity: sedentary, light, moderate, moderate-to-vigorous, and vigorous physical activity. The intensity of physical activity was established using the Evenson and colleagues’ [52] children algorithm cut-off points in the ActiLife software.

#### 2.4.7. Food Frequency Questionnaire (FFQ)

Participants completed a food frequency questionnaire developed by Matt and colleagues [53] to assess baseline differences in food intake between the EG and CG participants. Using this instrument, participants indicated how often they consumed food from the following food groups: fruits, vegetables, sweets, and sweetened beverages. Participants answered questions such as “How often did you eat/drink (food/beverage item)?” on a Likert-type scale: (1) never, (2) less than once a month, (3) once a month, (4) 1–2 per week, (5) 3–4 per week, (6) 5–6 per week, (7) 1 per day, and (8) 2 or more per day. This instrument was selected because of its improved intake recall and reporting in ethnically diverse populations versus prior instruments such as the Fred Hutchinson FFQ [53]. In this study, the FFQ internal consistency for the pre- and post-assessments was excellent (Cronbach’s α = 0.93 and 0.92, respectively).

### 2.5. Data Analysis

Statistical analyses for this study were conducted in four phases. First, the missing data pattern and statistical assumptions were analyzed using SPSS 23.0. The variables showed slight deviations from normality, with skewness ranging from 2.29 to 2.85 and kurtosis ranging from 11.02 to 0.97. According to the rules of thumb for normality from Hair et al. [54] and Byrne [55], skewness values should range between 2 and −2, and kurtosis between 7 and −7. Fortunately, repeated measures analysis of covariance (ANCOVA) is robust to violations of normality [56]. Second, we analyzed baseline descriptive characteristics for the retained and withdrawn children to assess potential bias based on study attrition. Then, we analyzed baseline descriptive characteristics for the EG and CG participants to determine baseline equivalence. Third, pre- and post-intervention changes in primary outcomes were analyzed using repeated measures ANCOVA with three covariates (gender, age, and BMI). ANCOVA assumptions (e.g., sphericity) were tested and no violations were detected. Fourth, the differential effects of the intervention based on the EG participant attendance (% of sessions attended) were explored using repeated measures ANCOVA with three covariates (gender, age, and BMI). Complete-case analysis was preferred over other approaches (e.g., multiple imputations of missing post-intervention scores) given the considerable number of outcome variables in relation to the modest sample size.

## 3. Results

### 3.1. Sample Size

**Figure 1:**
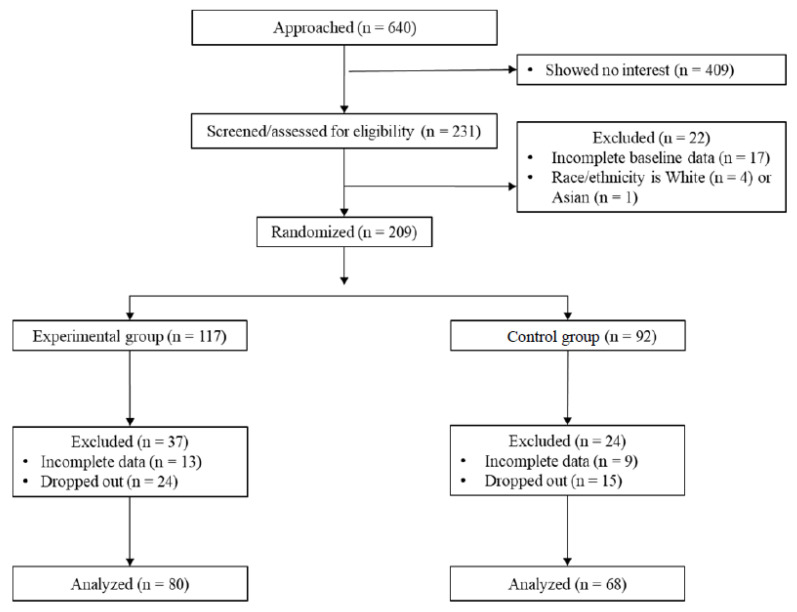
CONSORT Flow Diagram.

Initial recruitment efforts included reaching out to 640 Latinx and Black youth and their caregivers who received information about the study (Figure 1). Out of the contacted 640 parent–child dyads, 231 (36.1%) pairs expressed an interest in participating in the study orientation. At baseline, 17 participants dropped out and had incomplete data, and 5 participants were excluded for not meeting ethnicity requirements, thereby reducing the sample to 209 children. The remaining 209 children were randomly assigned to either the EG (*n* = 117) or CG (*n* = 92). After randomization, the final analytic sample was reduced to 148 participants due to 61 children (*n*_EG_ = 37, 31.6% of EG participants; *n*_CG_ = 24, 26.1% of CG participants) being excluded as a result of missing key post-intervention measures (*n*_EG_ = 13, *n*_CG_ = 9), or for dropping out of the study (*n*_EG_ = 24, *n*_CG_ = 15). Reasons for dropping out included children having time conflicts with competing academic and sports demands, school truancy, losing interest, and having unreliable transportation. Results from a sensitivity G*Power (F test, repeated measures ANOVA, within-between interaction) with the present sample size of *n* = 148, alpha error probability of 0.05, power of 0.8, and average correlation among repeated measures of 0.5 showed that a required effect size f of 0.12 is considered statistically significant.

Demographic and key variables of interest at baseline were compared between the remaining participants (*n* = 148; EG = 80, CG = 68) and those excluded from the analysis (*n* = 61). No significant group differences in demographic characteristics and study outcomes were found, except for sleep duration (Supplemental Tables S1 and S2). On average, total hours of sleep at night were significantly higher among the included participants (*M* = 8.61, *SD* = 2.10) than among the withdrawn children (*M* = 7.83, *SD* = 1.76; *t* (175) = 2.18, *p* = 0.03, and Cohen’s *d* = 0.39) [57].

### 3.2. Baseline Sample Descriptive Characteristics

#### 3.2.1. Demographic and Acculturation Characteristics

As shown in Table 1, most of the children were born in the United States (*n* = 134; 90.5%), self-identified as Latinx (*n* = 107; 72.3%), and reported a high acculturation level (*n* = 78; 74.3%). When comparing the EG and CG participants’ demographic characteristics, only ethnicity was significantly different between the groups (χ^2^(2, *n* = 107) = 8.83, *p* = 0.01, Cramer’s V = 0.24). That is, a larger percentage of Latinx participants were randomly assigned to the EG (78.8%) compared to CG (64.7%). No significant group differences at baseline were observed in participants’ age, gender, place of birth, and acculturation level, confirming the robustness of the randomization procedure.

#### 3.2.2. Weight Status and Percent Body Fat

As shown in Table 1, 52% of the participants were overweight or obese, 39.2% were of normal weight, 3.4% were underweight, and 5.4% missing. On average, participants had a percent body fat (%BF) of 27.47% (*SD* = 10.35%, values exceeding 75th percentile cutoffs of %BF for 10-year-old boys (24.5%) and 10-year-old girls (26.4%), respectively) [58]. We used 10-year-olds as a reference because the mean age of the sample was 10 years. There were no significant differences in weight status and %BF at baseline between the EG and CG participants.

#### 3.2.3. Emotional Eating, Anxiety, and Sleep Scores

As shown in Table 2, for emotional eating at baseline, participants had a mean composite emotional eating score of 1.69 (*SD* = 0.82), indicating some engagement in emotional eating. Participants’ T-scores on the MASC-2 averaged 51.43 (*SD* = 12.89), which falls in the average anxiety score range. Participants reported a mean global score of 3.86 (*SD* = 3.38) indicating, on average, good sleep quality. Specifically, participants reported a mean sleep duration of 8.61 h per night (*SD* = 2.10 h), had on average 88.24% sleep efficiency (*SD* = 14.28), and reported good sleep latency (scaled from 0—good to 3—bad; *M* = 0.71, *SD* = 0.85). No significant differences between the EG and CG participants were detected at baseline in emotional eating, anxiety, and some sleep variables such as global sleep quality, sleep duration, and sleep latency. However, sleep efficiency among the EG participants (*M* = 85.20, *SD* = 15.75) was significantly lower compared to that among the CG participants (*M* = 91.61, *SD* = 11.73; *t* (94) = −2.31, *p* = 0.02, Cohen’s *d* = 0.46).

#### 3.2.4. Physical Activity and Dietary Intake

At baseline, participants (*n* = 73) engaged in an average of 26.34 min (*SD* = 21.87 min) of daily moderate-to-vigorous physical activity (MVPA). In terms of their dietary intake, only 33% of participants at baseline reported consuming fruit and 12% consuming vegetables at least 5 times per day (data not tabled). Participants on average drank one sweetened beverage every day and ate sweets twice per day. There were no significant differences between the EG and CG participants regarding physical activity and dietary intake at baseline.

### 3.3. Intervention Effects on Emotional Eating, Anxiety, and Sleep Quality

#### 3.3.1. Emotional Eating, Anxiety, and Sleep Scores

Significant pre- and post-intervention effects on emotional eating and anxiety scores were observed among the EG participants, but not in the CG participants (Table 3). Specifically, there was a significant pre-to-post-intervention decrease in the EG participants’ emotional eating score (*F* (1,121) = 5.41, *p* = 0.02, partial *η*^2^ = 0.04), compared to that of CG participants. Likewise, there was a significant decrease in the EG participants’ anxiety total T-scores (*F* (1,118) = 5.09, *p* = 0.03, partial *η*^2^ = 0.04), separation anxiety/phobia subscale T-scores (*F* (1,117) = 12.53, *p* < 0.001, partial *η*^2^ = 0.10), and humiliation/rejection subscale T-scores (*F* (1,118) = 5.83, *p* = 0.02, partial *η*^2^ = 0.05) from T1 to T2. Regarding sleep quality, the EG and CG participants differed significantly in sleep latency (time taken to fall asleep) from pre- to post-intervention assessments (*F* (1,118) = 4.98, *p* = 0.03, partial *η*^2^ = 0.04, Table 3). On average, the EG participants improved their reported sleep latency pre- and post-intervention. By contrast, CG participants reported longer time needed to fall asleep both pre- and post-intervention. No significant differences in the rates of change were observed in sleep duration and sleep efficiency between the two treatment groups. It is noteworthy that both the EG and CG participants reported a good quality in terms of sleep duration, sleep efficiency, and sleep latency pre- and post-intervention.

#### 3.3.2. Physical Activity and Dietary Intake

We also assessed pre- and post-intervention differences in MVPA in the EG or CG participants (Table 3). On average, the EG participants (*n* = 31) recorded about 22 min per day of MVPA both before (*M* = 22.67, *SD* = 16.03) and after (*M* = 22.41, *SD* = 13.89) the intervention. CG participants (*n* = 33) decreased from an average of 29.44 min per day (*SD* = 26.30) at baseline to 21.49 min per day (*SD* = 21.49) after the intervention. In addition, no significant changes in dietary intake were observed pre- and post-intervention between the EG and CG participants (Table 3).

### 3.4. Intervention Dosage-Response Effects

Finally, we examined whether the changes in outcomes pre- and post-intervention for the EG participants were different depending on their intervention attendance by assessing a time-by-attendance interaction. As presented in Table 4, the results showed that after controlling for participants’ gender, age, and BMI, only the change in sleep efficiency across both time points differed significantly as a function of attendance level (*F* (1,18) = 2.61, *p* = 0.03, partial *η*^2^ = 0.67). This interaction indicates a greater increase in sleep efficiency among the EG participants with higher attendance rates. No other changes in outcomes were significantly different among the EG participants as a function of attendance. However, this finding might be explained by low variability in the EG participants’ attendance rate, as attendance was high among the EG participants; 97.6% of the EG participants attended at least 50% of the sessions (no tabulations are presented).

## 4. Discussion

This study tested the efficacy of a 12-week LUAM intervention in reducing emotional eating, reducing anxiety, and improving sleep quality in a sample of Latinx and Black youth using an RCT approach. To the authors’ knowledge, the LUAM intervention is one of the few RCT mindfulness studies assessing its impact on Latinx and Black early adolescents’ mental and physical health. Consistent with the study’s hypotheses, the findings of this research indicate that the LUAM intervention lowered pre- and post-intervention emotional eating in the EG participants when compared to those of CG participants. Our study’s effect of decreasing emotional eating scores in youth is consistent with previous research [59] and expands the extant understanding of the effect of mindfulness practices (e.g., mindful eating and breathing) on emotional eating in youth of color with overweight/obesity. The reduction in emotional eating through mindfulness practices is significant because mindfulness practices promote emotion regulation with the aim of reducing caloric intake [20] and sugar and fat consumption [21]. Mindful eating practices encourage youth to pay close attention to body-related sensations in response to the foods they consume as well as thoughts they have about food [22].

The results from the LUAM intervention also showed positive effects on lowering anxiety and sleep disturbance scores in Latinx and Black youth after participation in the EG compared to the CG. Our findings are congruent with several studies that have shown mindfulness practices (e.g., meditation and breathing) as being helpful in reducing anxiety and sleep disturbances among children and adolescents [5,6,26,27,28]. In addition, the use of a mindfulness–exercise-based approach (e.g., yoga and spinning/kickboxing) supports research suggesting that such approaches are negatively associated with both anxiety [60,61,62] and sleep disturbances [37,38]. Overall, given the connection between sleep problems and the development of psychological distress including anxiety and depression [62,63,64], our findings also provide a promising approach for lowering anxiety and sleep disturbances.

It is noteworthy to mention that the LUAM intervention did not show significant changes in pre- and post-intervention daily minutes of MVPA between the EG and CG participants. It would seem plausible to suggest that the exercise dosage in the current study was not sufficient to see a marked increase in daily time spent in MVPA. Although results among youth tend to be mixed, there is some evidence to suggest that there may be physical activity compensation among youth. That is, youth who engage in increased physical activity on a particular day may compensate for this activity by decreasing their activity the following day. Indeed, among a group of 8–11-year-olds, Ridgers et al. [65] found that when participants engaged in higher levels of light physical activity (LPA), they engaged in less LPA and MVPA the following day. Likewise, increased MVPA time was associated with less LPA and MVPA time the following day. Thus, it could be that youth in the current study may have been compensating for the activity received as part of the intervention during non-intervention times. However, more research is needed to test this hypothesis. Another potential reason that there were no statistically significant differences in pre-to-post-intervention daily minutes of MVPA between the EG and CG participants is the relatively low amount of complete data in each group. It is possible that those participants in both groups who wore the accelerometer had greater awareness of their levels of physical activity and thus may have engaged in physical activity outside of the intervention at relatively similar levels.

### Limitations

This study has several limitations. First, because of the small and unequal sample size of Latinx and Black participants, we were unable to examine the impact of this intervention on participants by ethnicity. Second, because neither the Latinx nor Black populations are monolithic, nor are their social environments and family contexts, our results might not be generalizable to a wider population of Latinx and Black youth. Participants in this study included mostly Latinx youth born in the United States, with high acculturation levels. Future studies should include a larger representative sample of Latinx and Black youth to be able to disaggregate the sample and to examine biopsychosocial factors that might differentially impact the intervention effects across the two ethnic groups. Some of these biopsychosocial factors could include parent stressors related to safety in the community, which have been positively associated with obesity among adolescents [66]. Third, although the PSQI is frequently used as a measure of sleep quality, the relatively low internal consistency for the current study should be noted. Alternative sleep quality measures should be considered in the future. Fourth, participants in both the EG and CG were recruited from the same set of schools. Despite the random assignment, it is possible that participants who were randomly assigned to the EG discussed the activities in which they engaged as part of the intervention which may have inadvertently impacted the CG participants (e.g., the CG participants may have become more aware of their physical activity, eating, and sleeping habits because of learning about the EG intervention). However, differences between the EG and CG participants were still identified, indicating that the mindfulness–exercise-based programs may have positively impacted the youth. Fifth, although randomization minimizes the potential for bias as it enables comparisons to be made between the experimental and control groups, it is possible that other biases, such as performance bias, may have been present among the EG participants. The performance bias might be due to differences between groups in the treatments that they receive, or in exposure to factors other than the interventions of interest. Sixth, this study examined the immediate impact of the intervention on youth. It is unknown whether these changes are sustained long-term. For example, it is unclear whether youth in the EG continued to engage in mindfulness practices once the 12-week program was complete. It is also possible that the positive effects of the intervention might be short-lived and inconsequential in the long term if some family factors such as parental stressors are not addressed. Future studies should examine the sustained, longer-term impact of the intervention and how this long-term impact could be hindered by family factors.

## 5. Conclusions

Despite these limitations, this study provides preliminary evidence, through an RCT approach, that there are benefits to utilizing mindfulness practices (e.g., mindful eating and exercise-based approaches) in reducing emotional eating and anxiety and increasing sleep quality among youth of color. Given that there have been relatively few studies examining the impact of mindfulness interventions, especially among marginalized youth who are at an increased risk for obesity, this study begins to fill an important gap in the literature addressing the psychological factors associated with obesity. Further, apart from spinning, the activities and practices included in the LUAM intervention (i.e., kickboxing and mindful eating) can be completed without equipment, at youths’ homes. This has important implications for the feasibility of such an intervention in a real-world setting.

## Figures and Tables

**Table 1:** Baseline sample descriptive characteristics (*N* = 148, *n*_EG_ = 80, *n*_CG_ = 68).

				Comparison of Experimental and Control Groups					
Variables	Total Sample	Experimental Group	Control Group	*df*	*t*	*χ* ^2^	*p*	*d* ^a^	Cramer’s V
Age in years, *M* (*SD*)	10.11 (1.3)	9.93 (1.2)	10.34 (1.4)	146	1.90		0.06	0.31	
	*N* (*%*)								
Gender				1		0.21	0.65		0.04
Boy	71 (48.0)	37 (46.3)	34 (50.0)						
Girl	77 (52.0)	43 (53.7)	34 (50.0)						
Ethnicity				2		8.83	0.01		0.24
Latinx	107 (72.3)	63 (78.8)	44 (64.7)						
Black	28 (18.9)	15 (18.8)	13 (19.1)						
Multiracial/Other	13 (8.8)	2 (2.4)	11 (16.2)						
Place of birth				3		4.02	0.26		0.17
U.S.	134 (90.5)	74 (92.4)	60 (88.2)						
Mexico	8 (5.4)	3 (3.8)	5 (7.4)						
Central America	2 (1.4)	0 (0.0)	2 (2.9)						
Other	4 (2.7)	3 (3.8)	1 (1.5)						
Acculturation				1		0.28	0.60		0.05
High	78 (74.3)	45 (76.3)	33 (71.7)						
Low	27 (25.7)	14 (23.7)	13 (28.3)						
Obesity status				3		0.66	0.88		0.07
Underweight	5 (3.4)	3 (3.8)	2 (2.9)						
Normal weight	58 (39.2)	31 (38.8)	27 (39.7)						
Overweight	24 (16.2)	11 (13.8)	13 (19.1)						
Obese	53 (35.8)	29 (36.2)	24 (35.3)						
Missing	8 (5.4)	6 (7.5)	2 (2.9)						
Percent body fat	27.55 (10.5)	27.31 (11.0)	27.81 (10.0)	138	0.28		0.78	0.05	
Percent attendance, *M* (*SD*)	84.83 (14.3)	86.22 (11.9)	83.22 (16.7)	145	1.27		0.21	0.21	

Note. ^a^ Cohen’s *d*.

**Table 2:** Comparison of baseline means and standard deviations of key variables between the experimental and control groups.

	Total Sample		Experimental Group		Control Group			
Variables	*N*	*M* (*SD*)	*N*	*M* (*SD*)	*N*	*M* (*SD*)	*t*-Test	*d* ^b^
Emotional eating score	142	1.69 (0.82)	78	1.71 (0.80)	64	1.65 (0.86)	0.43	0.07
Total anxiety *T*-score	141	51.43 (12.89)	77	51.95 (13.14)	64	50.80 (12.64)	0.53	0.09
Separation anxiety/phobia	141	53.04 (11.35)	77	53.40 (11.07)	64	52.61 (11.75)	0.41	0.07
Generalized anxiety disorder	141	49.89 (12.87)	77	50.04 (12.39)	64	49.72 (13.52)	0.15	0.02
Social anxiety	141	48.67 (11.69)	77	49.45 (11.80)	64	47.73 (11.59)	0.87	0.15
Humiliation/rejection	141	48.12 (11.16)	77	49.17 (11.19)	64	46.86 (11.08)	1.23	0.21
Performance fears	141	49.91 (12.41)	77	50.16 (12.73)	64	49.61 (12.11)	0.26	0.04
Obsessions and compulsions	141	55.02 (11.70)	77	55.49 (11.94)	64	54.47 (11.48)	0.51	0.08
Physical symptoms	141	52.40 (11.83)	77	52.62 (11.14)	64	52.14 (12.68)	0.24	0.04
Panic	141	53.13 (12.72)	77	53.35 (12.04)	64	52.86 (13.59)	0.23	0.04
Tense and restless	141	51.30 (10.78)	77	51.52 (10.36)	64	51.03 (11.35)	0.27	0.04
Harm avoidance	141	44.55 (12.22)	77	43.99 (12.28)	64	45.23 (12.21)	−0.60	0.10
Global sleep quality	148	3.86 (3.38)	80	4.09 (3.18)	68	3.60 (3.61)	0.87	0.14
Sleep duration (hours)	136	8.61 (2.10)	74	8.57 (1.99)	62	8.67 (2.23)	−0.28	0.05
Sleep efficiency (%)	99	88.24 (14.28)	52	85.20 (15.75)	47	91.61 (11.73)	−2.31 *	0.46
Sleep latency	140	0.71 (0.85)	76	0.79 (0.93)	64	0.61 (0.75)	1.25	0.21
Daily minutes MVPA ^a^	73	26.34 (21.87)	39	24.10 (17.44)	34	28.90 (26.09)	−0.94	0.22
Dietary intake (servings/day)								
Fruits	92	4.85 (5.19)	53	5.35 (4.92)	39	4.18 (5.53)	1.07	0.23
Vegetables	92	2.76 (4.41)	53	2.16 (2.33)	39	3.57 (6.17)	−1.35	0.32
Sweets	92	2.00 (3.18)	53	2.27 (3.34)	39	1.64 (2.94)	0.94	0.20
Sweetened beverages	142	0.77 (1.09)	78	0.70 (1.06)	64	0.86 (1.14)	−0.87	0.15

Note. ^a^ MVPA = moderate-to-vigorous physical activity. ^b^ Cohen’s *d*, * *p* < 0.05.

**Table 3:** Pre- and post-intervention changes in outcome variables between the experimental and control groups.

	Experimental Group			Control Group			Time-by-Group Interaction	
Variables	*N*	Pre (*M*, *SD*)	Post (*M*, *SD*)	*N*	Pre (*M*, *SD*)	Post (*M*, *SD*)	*F*	Partial *η*^2^
Emotional eating score	71	1.70 (0.81)	1.49 (0.65)	55	1.59 (0.84)	1.88 (0.93)	5.41 *	0.04
Total anxiety *T*-score	69	52.70 (13.42)	47.33 (11.82)	54	49.96 (12.86)	49.83 (13.02)	5.09 *	0.04
Separation anxiety/phobia	69	54.14 (10.93)	48.62 (11.15)	53	52.38 (12.06)	53.79 (12.41)	12.53 **	0.10
Generalized anxiety disorder	69	50.36 (12.64)	46.33 (10.84)	54	48.67 (13.81)	48.56 (13.70)	2.54	0.02
Social anxiety	69	49.67 (12.13)	44.77 (9.89)	54	46.69 (11.65)	45.91 (12.00)	3.27	0.03
Humiliation/rejection	69	49.51 (11.26)	44.23 (9.86)	54	45.62 (11.22)	45.20 (11.50)	5.83 *	0.05
Performance fears	70	50.26 (13.07)	47.31 (10.42)	54	49.02 (12.17)	48.17 (11.62)	0.17	0.00
Obsessions and compulsions	70	56.50 (11.84)	53.61 (11.57)	54	53.81 (11.85)	54.41 (13.17)	1.43	0.01
Physical symptoms	69	53.29 (11.28)	51.12 (10.00)	54	51.41 (12.68)	51.98 (12.42)	0.97	0.01
Panic	69	53.93 (12.31)	51.55 (11.57)	54	51.89 (13.71)	52.26 (12.43)	0.82	0.01
Tense and restless	69	52.22 (10.40)	50.43 (9.13)	54	50.70 (11.33)	51.69 (12.25)	1.05	0.01
Harm avoidance	69	43.96 (11.91)	39.55 (14.20)	54	45.56 (12.47)	41.78 (15.92)	0.51	0.00
Sleep quality								
Sleep duration (hours)	62	8.60 (1.93)	8.37 (2.03)	47	8.51 (1.83)	8.63 (1.48)	1.71	0.02
Sleep efficiency (%)	36	86.12 (16.63)	89.39 (18.80)	29	93.16 (11.80)	91.72 (11.80)	0.11	0.00
Sleep latency	69	0.81 (0.91)	0.70 (0.86)	54	0.50 (0.67)	0.67 (0.78)	4.98 *	0.04
Daily minutes MVPA ^a^	31	22.67 (16.03)	22.41 (13.89)	33	29.44 (26.30)	21.49 (12.45)	1.72	0.03
Dietary intake (servings/day)								
Fruits	53	5.35 (4.92)	4.78 (5.50)	36	3.99 (5.49)	3.94 (4.81)	0.31	0.00
Vegetables	53	2.16 (2.33)	2.96 (4.50)	36	2.75 (4.81)	2.41 (3.87)	1.56	0.02
Sweets	53	2.27 (3.34)	1.55 (1.97)	36	1.37 (2.20)	1.90 (2.99)	3.29	0.04
Sweetened beverages	71	0.67 (1.08)	0.71 (0.89)	56	0.79 (1.09)	0.83 (1.15)	0.09	0.00

Note. ^a^ MVPA = moderate-to-vigorous physical activity, * *p* < 0.05, ** *p* < 0.001.

**Table 4:** Pre- and post-intervention changes in outcome variables in the experimental group as a function of attendance.

		*M* (*SD*)		Time-by-Attendance Interaction	
Variables	*N*	Pre	Post	*F*	Partial *η*^2^
Emotional eating score	70	1.71 (0.81)	1.48 (0.65)	0.51	0.19
Anxiety total *T*-score	68	52.62 (13.50)	47.13 (11.78)	1.05	0.32
Separation anxiety/phobia	68	54.13 (11.01)	48.68 (11.22)	0.75	0.25
Generalized anxiety disorder	68	50.41 (12.72)	46.01 (10.59)	1.20	0.35
Social anxiety	68	49.59 (12.20)	44.59 (9.85)	1.49	0.40
Humiliation/rejection	68	49.41 (11.31)	44.06 (9.82)	0.99	0.31
Performance fears	69	50.23 (13.17)	47.19 (10.44)	1.74	0.45
Obsessions and compulsions	69	56.29 (11.80)	53.38 (11.49)	1.53	0.42
Physical symptoms	68	53.18 (11.33)	50.97 (10.00)	0.45	0.17
Panic	68	53.76 (12.33)	51.28 (11.33)	0.46	0.17
Tense and restless	68	52.19 (10.47)	50.47 (9.19)	0.69	0.24
Harm avoidance	68	44.09 (11.94)	39.32 (14.17)	0.76	0.26
Sleep quality					
Sleep duration (hours)	62	8.60 (1.93)	8.37 (2.03)	0.96	0.34
Sleep efficiency (%)	36	86.12 (16.63)	89.39 (18.80)	2.61 *	0.67
Sleep latency	69	0.81 (0.91)	0.70 (0.86)	0.75	0.26
Daily minutes MVPA ^a^	30	22.70 (16.31)	22.52 (14.11)	0.90	0.36
Daily dietary intake (servings/day)					
Fruits	52	5.18(4.80)	4.67 (5.50)	1.71	0.46
Vegetables	52	2.09 (2.28)	3.01 (4.53)	0.92	0.32
Sweets	52	2.15 (3.26)	1.51 (1.97)	0.65	0.24

Note. ^a^ = MVPA = moderate-to-vigorous physical activity, * *p* < 0.05.

## Data Availability

The data presented in this study are available upon request from the corresponding author, and five years upon completion of the grant. The data are not publicly available due to concerns regarding privacy.

## Supplementary Materials

The following supporting information can be downloaded at: https://www.mdpi.com/article/10.3390/children10030538/s1, Table S1. Comparison of Sample Descriptive Characteristics at Baseline Between the Retained and Excluded Participants (N_final = 148, N_excluded = 61); Table S2. Comparison of Outcome Measures at Baseline Between the Retained and Excluded Participants After Randomization (N_final = 148, N_excluded = 61).
