# Supplementary material for: Lace Up and Mindfulness: A Randomized Controlled Trial Intervention to Reduce Emotional Eating, Anxiety, and Sleep Disturbances in Latinx and Black Youth
Source: Children (Basel). 2023 Mar 10;10(3):538. doi: 10.3390/children10030538 (PMC10046922; doi:10.3390/children10030538)
Supplement: Supplementary file 1 [file children-10-00538-s001.zip › children-2237963-supplementary.pdf]

Supplemental Tables S1. Comparison of Sample Descriptive Characteristics at Baseline Between the Retained and Excluded Participants (N_final = 148, N_excluded = 61).

Variables	Retained	Excluded	*df*	*t*	χ^2	*p*	Cohen's *d*	Cramer 's V
Age in years, *M* (*SD*)	10.11 (1.33)	10.49 (1.46)	207	-1.81		.07	0.28	
	N (%)							
Gender			1		0.07	.79		.02
Boy	71 (48.0)	30 (50.0)						
Girl	77 (52.0)	30 (50.0)						
Ethnicity			2		5.9	.05*		.17
Latinx	107 (72.3)	35 (60.3)						
Black	28 (18.9)	20 (34.5)						
Multiracial/ Other	13 (8.8)	3 (5.2)						
Place of birth			3		5.44	.14		.17
U.S.	134 (90.5)	47 (95.9)						
Mexico	8 (5.4)	0 (0.0)						
Central America	2 (1.4)	2 (1.4)						
Other	4 (2.7)	0 (0.0)						
Acculturation			1		0.95	.33		.08
High	78 (74.3)	32 (82.1)						
Low	27 (25.7)	7 (17.9)						
Obesity status			3		2.66	.45		.12
Underweight	5 (3.4)	0 (0.0)						
Normal weight	58 (39.2)	23 (37.7)						
Overweight	24 (16.2)	8 (13.1)						
Obese	53 (35.8)	25 (41.0)						
Unreported	8 (5.4)	3 (4.9)						
Percent body fat	27.55 (10.5)	29.40 (13.1)	89	-0.96		.34	0.16	

* p < .05.

Supplemental Tables S2. Comparison of Outcome Measures at Baseline Between the Retained and Excluded Participants After Randomization (N_final = 148, N_excluded = 61).

	Retained		Excluded			
Variables	*N*	*M (SD)*	*N*	*M (SD)*	*t*-test	Cohen's *d*
Emotional eating	142	1.69 (0.82)	44	1.88 (0.92)	-1.34	0.23
Anxiety total *T*-score	141	51.43 (12.89)	41	50.61 (12.22)	0.36	0.06
Separation anxiety/phobia	141	53.04 (11.35)	41	50.46 (11.72)	1.27	0.23
Generalized anxiety disorder	141	49.89 (12.87)	41	49.00 (11.11)	0.40	0.07
Social anxiety	141	48.67 (11.69)	41	48.12 (10.09)	0.27	0.04
Humiliation/rejection	141	48.12 (11.16)	41	47.71 (10.89)	0.21	0.03
Performance fears	141	49.91 (12.41)	41	48.59 (13.01)	0.59	0.11
Obsessions and compulsions	141	55.02 (11.70)	41	55.39 (13.12)	-0.17	0.03
Physical symptoms	141	52.40 (11.83)	41	53.41 (10.33)	-0.50	0.08
Panic	141	53.13 (12.72)	41	54.78 (12.27)	-0.74	0.13
Tense and restless	141	51.30 (10.78)	41	51.49 (10.26)	-0.10	0.02
Harm avoidance	141	44.55 (12.22)	41	43.24 (13.09)	0.59	0.11
Sleep quality	148	3.86 (3.38)	61	3.46 (3.73)	0.77	0.12
Sleep duration (hours)	136	8.61 (2.10)	41	7.83 (1.76)	2.18*	0.39
Sleep efficiency (%)	99	88.24 (14.28)	32	85.35 (14.98)	0.98	0.20
Sleep latency	140	0.71 (0.85)	44	0.82 (0.79)	-0.77	0.13
Daily minutes MVPA[a]	73	26.34 (21.87)	28	34.61 (44.92)	-1.24	0.28
Daily dietary intake (servings/day)						
Fruits	92	4.85 (5.19)	27	5.20 (5.02)	-0.31	0.07
Vegetables	92	2.76 (4.41)	29	4.07 (4.99)	-1.35	0.29
Sweets	92	2.00 (3.18)	27	2.39 (3.38)	-0.54	0.12
Sweetened beverages	142	0.77(1.09)	46	1.06 (0.87)	-1.60	0.27
Percent body fat	140	27.55 (10.47)	58	29.40 (13.06)	-0.96	0.07

Note. aMVPA = moderate-to-vigorous physical activity.
* p < .05.
